# Is Electricity Beneficial in Extracting Teeth?

**Published:** 1859-06

**Authors:** O. W. True

**Affiliations:** Elm Tree Farm, Maine


### IS ELECTRICITY BENEFICIAL IN EXTRCTING TEETH?

BY O. W. TRUE.

PERADVENTURE the subject of electricity has been fully treated upon in the "*Register*," and more, at this time, upon the subject will be *ad nauseam;* but as I have had the privilege of seeing or reading only the last March number, permit me, then, to have a friendly word with you about its uses, as it is and has been applied to surgical operations upon the teeth, etc., at the present day.

Although, of however little importance the having a tooth extracted may seem to be to one who has been fortunate enough to escape this ordeal; or, whatever amount of jest may be cast upon the seemingly timid one—who is truly a sensitive and fine attuned unfortunate one; whose nerves of sensation already, as it were, stand out in the front rank in acute sensitiveness, it is all of naught to them at this particular time. For the good of this latter and truly valuable class, various expedients have been resorted to, among which anæsthetics hold a prominent position.

But that serious accidents are liable to follow the administration of the powerful anæsthetics which are now in use, none will deny, it is presumed, when even they are administered under the guidance of those master minds whose hands are skilled in their use. Hence, if an agent, although it may be the elements of the lightning, can be chained, and tamed, and made to administer to the alleviation of a single pang of the sorrows arising to poor diseased fellow-being, which shall be at the same time safe, efficient, and applicable, even though it should be only in minor cases, it should be duly hailed with a welcome embrace in every land.

But, just now, a glad sound is heard going forth in honor of electricity. Whether this meed belongs to it or no, time and experience—unerring judges—will truly show. But still, its earnest cry, should it become "zealous for its reputation as a therapeutic agent," would be, I fear, could it articulate its rejoinder, "save me from my friends."

Although it has been but a few years since it was rescued from comparative obscurity, by such men as Golding, Bird, Duchenne, and others of like-minded acumen, whose praiseworthy efforts are ever remembered with gratitude in all coming time. But notwithstanding this honorable introduction, the unqualified laudations which are bestowed upon it from some quarters sounds rather quack-like, and is not likely to secure it so lasting a place among our remedial resources.

But let us refer to experience and tests, and those, too, conducted by able and scientific experimenters, as we pass along, to aid in judging of the really beneficial effects, which may be reasonably looked for by calling electricity to our aid, while waiting patiently for the further unraveling of the difficult question now before us.

That this question will be justly and satisfactorily decided ere long, we need but look at the advances made to satisfy ourselves; while remembering that the Greek and Roman physicians had to resort to the electric fishes for electric machines, while we have electric machines that are an ornament, if not of use, in the most fastidious lady's *boudior*, with "to-and-fro," and friend S. B. Smith's "direct" current.

Dr. Hiffelsheim's experience in thirty-six cases of neuralgia, with electricity, has been "highly satisfactory" to him. Dr. Althus publishes some of his experience, in the *Medical Times and Gazette*, which are also satisfactory. But Dr. B. W. Richardson, after submitting himself to careful and painful experiments, he arrived at this result, "that the electric current can not, according to our present knowledge of its application, be made practicable for the production of local anæsthesia."

Dr. Althus, replying to him, says: "When the current is applied in a different manner, the sensibility is notably diminished." What is this "different manner?" But Dr. Althus goes on to state further, that "the result is much more striking if there is a morbid increase of sensibility in a nerve, as in neuralgia, than if a nerve, in its normal state, is acted upon." While Mr. Henry W. Lobb says: "I have never used it to prevent pain during the extraction of teeth." Why not? Are you slow to travel "where angels fear to tread?" But he goes on to say, "but from what I know of its success in toothache, the following plan will, no doubt, be found perfectly successful. Procure a 60 link—" etc., etc. Hence, Mr. Eden, of Brighton, did "procure a 60 link, etc., etc.," and thence, "followed minutely the directions" of Mr. Lobb, but he was "sorry to say" that even then he "did not obtain any diminution of sensibility."

A reviewer in *The Brit. and For. Med. Chi. Review*, after carefully analyzing the evidence for and against the use of electricity, says: "It appears that hyperæsthesia may be reduced;" (that is, the question of "Electrical Anæsthesia,") "and even that the normal sensibility may be diminished; but there is no evidence that the reduction can be carried so far as to render electricity a useful anæsthetic agent."

I intended to have given some more experiments, particularly upon the extraction of teeth by the aid of electricity, but must defer them till another time.

ELM TREE FARM, Maine.

